# Supplementary material for: Modeling glioblastoma heterogeneity as a dynamic network of cell states
Source: Mol Syst Biol. 2021 Sep 16;17(9):e10105. doi: 10.15252/msb.202010105 (PMC8444284; doi:10.15252/msb.202010105)
Supplement: Supplementary file 6 — Source Data for Figure 5 [file MSB-17-e10105-s004.zip › Figure5A_sourcedata/GSEA_3017/nosigdb_stateC.GseaPreranked.1622795002785/RICHARDS_NOWAKOWSKI_SCIENCE_2017_MURAL_UPREG.html]

Details for gene set RICHARDS\_NOWAKOWSKI\_SCIENCE\_2017\_MURAL\_UPREG[GSEA]

|  || Dataset | state13017 |
| Phenotype | NoPhenotypeAvailable |
| Upregulated in class | na\_neg |
| GeneSet | RICHARDS\_NOWAKOWSKI\_SCIENCE\_2017\_MURAL\_UPREG |
| Enrichment Score (ES) | -0.429056 |
| Normalized Enrichment Score (NES) | -2.8149493 |
| Nominal p-value | 0.0 |
| FDR q-value | 0.0 |
| FWER p-Value | 0.0 |
Table: GSEA Results Summary

  

Fig 1: Enrichment plot: RICHARDS\_NOWAKOWSKI\_SCIENCE\_2017\_MURAL\_UPREG      
 Profile of the Running ES Score & Positions of GeneSet Members on the Rank Ordered List

  

| PROBE | GENE SYMBOL | GENE\_TITLE | RANK IN GENE LIST | RANK METRIC SCORE | RUNNING ES | CORE ENRICHMENT || 1 | KLHL23 |  |  | 84 | 0.359 | -0.1509 | No |
| 2 | SLC12A2 |  |  | 148 | 0.288 | -0.2634 | No |
| 3 | CAST |  |  | 205 | -0.253 | -0.3636 | No |
| 4 | IGFBP7 |  |  | 225 | -0.259 | -0.3920 | No |
| 5 | FERMT2 |  |  | 227 | -0.259 | -0.3855 | No |
| 6 | CLIC1 |  |  | 237 | -0.261 | -0.3945 | No |
| 7 | LIMS1 |  |  | 244 | -0.262 | -0.3977 | No |
| 8 | TPT1 |  |  | 248 | -0.263 | -0.3950 | No |
| 9 | MYOF |  |  | 251 | -0.263 | -0.3903 | No |
| 10 | AGTRAP |  |  | 257 | -0.266 | -0.3914 | No |
| 11 | PPIB |  |  | 265 | -0.268 | -0.3963 | No |
| 12 | RPL41 |  |  | 270 | -0.269 | -0.3954 | No |
| 13 | MEG3 |  |  | 273 | -0.270 | -0.3905 | No |
| 14 | EHD2 |  |  | 275 | -0.270 | -0.3837 | No |
| 15 | RRBP1 |  |  | 283 | -0.271 | -0.3885 | No |
| 16 | RPL23 |  |  | 288 | -0.273 | -0.3875 | No |
| 17 | PHLDA1 |  |  | 295 | -0.274 | -0.3902 | No |
| 18 | SLC1A3 |  |  | 307 | -0.281 | -0.4024 | No |
| 19 | NR2F2 |  |  | 313 | -0.283 | -0.4030 | No |
| 20 | PLEKHH2 |  |  | 319 | -0.284 | -0.4035 | No |
| 21 | SERPINE1 |  |  | 323 | -0.286 | -0.4000 | No |
| 22 | SPATS2L |  |  | 339 | -0.290 | -0.4197 | Yes |
| 23 | HEY2 |  |  | 341 | -0.293 | -0.4122 | Yes |
| 24 | PPP1R15A |  |  | 342 | -0.293 | -0.4027 | Yes |
| 25 | DLC1 |  |  | 347 | -0.296 | -0.4009 | Yes |
| 26 | CHST2 |  |  | 350 | -0.297 | -0.3952 | Yes |
| 27 | COL4A2 |  |  | 353 | -0.300 | -0.3894 | Yes |
| 28 | ITGB5 |  |  | 356 | -0.300 | -0.3835 | Yes |
| 29 | S100A11 |  |  | 361 | -0.301 | -0.3815 | Yes |
| 30 | SPRY1 |  |  | 369 | -0.307 | -0.3852 | Yes |
| 31 | PMEPA1 |  |  | 375 | -0.308 | -0.3849 | Yes |
| 32 | LMNA |  |  | 376 | -0.308 | -0.3750 | Yes |
| 33 | FOS |  |  | 377 | -0.309 | -0.3650 | Yes |
| 34 | RHOB |  |  | 391 | -0.316 | -0.3800 | Yes |
| 35 | PLTP |  |  | 397 | -0.319 | -0.3793 | Yes |
| 36 | TIMP1 |  |  | 399 | -0.320 | -0.3709 | Yes |
| 37 | ZFP36L2 |  |  | 401 | -0.321 | -0.3625 | Yes |
| 38 | CALD1 |  |  | 420 | -0.335 | -0.3865 | Yes |
| 39 | SLC2A3 |  |  | 422 | -0.336 | -0.3776 | Yes |
| 40 | KLF6 |  |  | 429 | -0.339 | -0.3783 | Yes |
| 41 | WWTR1 |  |  | 437 | -0.345 | -0.3807 | Yes |
| 42 | WLS |  |  | 438 | -0.345 | -0.3695 | Yes |
| 43 | FAT1 |  |  | 441 | -0.346 | -0.3622 | Yes |
| 44 | HSPA5 |  |  | 447 | -0.350 | -0.3606 | Yes |
| 45 | MYL12A |  |  | 448 | -0.350 | -0.3493 | Yes |
| 46 | DKK3 |  |  | 454 | -0.356 | -0.3475 | Yes |
| 47 | RHOC |  |  | 455 | -0.359 | -0.3359 | Yes |
| 48 | B2M |  |  | 468 | -0.371 | -0.3471 | Yes |
| 49 | HES1 |  |  | 470 | -0.373 | -0.3370 | Yes |
| 50 | ADAM12 |  |  | 472 | -0.374 | -0.3269 | Yes |
| 51 | HBEGF |  |  | 480 | -0.386 | -0.3280 | Yes |
| 52 | IFITM2 |  |  | 483 | -0.388 | -0.3193 | Yes |
| 53 | VCL |  |  | 484 | -0.388 | -0.3068 | Yes |
| 54 | ENDOD1 |  |  | 493 | -0.405 | -0.3092 | Yes |
| 55 | LDHA |  |  | 498 | -0.412 | -0.3036 | Yes |
| 56 | JUN |  |  | 503 | -0.425 | -0.2977 | Yes |
| 57 | F3 |  |  | 505 | -0.432 | -0.2856 | Yes |
| 58 | PRSS23 |  |  | 510 | -0.438 | -0.2792 | Yes |
| 59 | SYTL2 |  |  | 513 | -0.439 | -0.2689 | Yes |
| 60 | CD9 |  |  | 515 | -0.443 | -0.2566 | Yes |
| 61 | PTN |  |  | 517 | -0.446 | -0.2441 | Yes |
| 62 | CA2 |  |  | 518 | -0.448 | -0.2297 | Yes |
| 63 | CEBPD |  |  | 523 | -0.455 | -0.2227 | Yes |
| 64 | TNFRSF21 |  |  | 526 | -0.461 | -0.2117 | Yes |
| 65 | IFITM3 |  |  | 533 | -0.477 | -0.2079 | Yes |
| 66 | COL6A1 |  |  | 534 | -0.479 | -0.1925 | Yes |
| 67 | VMP1 |  |  | 542 | -0.502 | -0.1898 | Yes |
| 68 | COL4A1 |  |  | 545 | -0.507 | -0.1773 | Yes |
| 69 | PLAT |  |  | 556 | -0.538 | -0.1793 | Yes |
| 70 | SPARCL1 |  |  | 557 | -0.544 | -0.1617 | Yes |
| 71 | ANXA2 |  |  | 563 | -0.567 | -0.1531 | Yes |
| 72 | MT2A |  |  | 566 | -0.575 | -0.1384 | Yes |
| 73 | SPARC |  |  | 567 | -0.575 | -0.1199 | Yes |
| 74 | LAMA4 |  |  | 571 | -0.620 | -0.1057 | Yes |
| 75 | ITPR2 |  |  | 572 | -0.634 | -0.0852 | Yes |
| 76 | CD44 |  |  | 575 | -0.659 | -0.0678 | Yes |
| 77 | NEAT1 |  |  | 579 | -0.683 | -0.0515 | Yes |
| 78 | ATP1B2 |  |  | 585 | -0.714 | -0.0382 | Yes |
| 79 | PMP22 |  |  | 590 | -0.837 | -0.0189 | Yes |
| 80 | LPL |  |  | 593 | -0.885 | 0.0058 | Yes |
Table: GSEA details [plain text format]

  

Fig 2: RICHARDS\_NOWAKOWSKI\_SCIENCE\_2017\_MURAL\_UPREG: Random ES distribution      
 Gene set null distribution of ES for **RICHARDS\_NOWAKOWSKI\_SCIENCE\_2017\_MURAL\_UPREG**
